# SLIT/ROBO Pathway and Prostate Cancer: Gene and Protein Expression and Their Prognostic Values

**DOI:** 10.3390/ijms26115265

**Published:** 2025-05-30

**Authors:** Nilton J. Santos, Francielle C. Mosele, Caroline N. Barquilha, Isabela C. Barbosa, Flávio de Oliveira Lima, Guilherme Oliveira Barbosa, Hernandes F. Carvalho, Flávia Karina Delella, Sérgio Luis Felisbino

**Affiliations:** 1 Laboratory of Extracellular Matrix Biology, Department of Structural and Functional Biology, Institute of Biosciences of Botucatu (IBB), São Paulo State University (UNESP), Botucatu 18618-689, São Paulo, Brazil; nilton.unesp@gmail.com (N.J.S.); francmosele@gmail.com (F.C.M.); caroline.barquilha@gmail.com (C.N.B.); gioeisabela@gmail.com (I.C.B.); flavia.delella@unesp.br (F.K.D.); 2 Laboratory of Extracellular Matrix and Gene Regulation, Department of Structural and Functional Biology, Institute of Biology (IB), University of Campinas (UNICAMP), Campinas 13083-865, São Paulo, Brazil; hern@unicamp.br; 3 Laboratory of Pathology, State Hospital of Bauru, Bauru 17033-360, São Paulo, Brazil; 4 Paracrine Signaling in Tissue Organization Laboratory, Institute of Biology (IB), University of Campinas (UNICAMP), Campinas 13083-865, São Paulo, Brazil; guiolive@unicamp.br

**Keywords:** prostate cancer, SLIT, ROBO, extracellular matrix, Pten, p53, *knockout* mice, RNA-seq

## Abstract

Prostate cancer (PCa) is the second most common cancer and the second leading cause of cancer-related mortality among men. Gene expression analysis has been crucial in understanding tumor biology and providing disease progression markers. Cell surface glycoproteins and those in the extracellular matrix play significant roles in the PCa microenvironment by promoting migration, invasion, and metastasis. The molecular and histopathological heterogeneity of prostate tumors necessitates a new marker discovery to better stratify patients at risk for poor prognosis. In this study, our objectives were to investigate and characterize the localization and expression of SLIT/ROBO in PCa samples from transgenic mice and human tumor samples, aiming to identify novel prognostic markers and potential therapeutic targets. We conducted histopathological, immunohistochemical, and bioinformatics analyses on prostate tumors from two *knockout* mice models (*Pb-Cre4/Pten^f/f^* and *Pb-Cre4/Trp53^f/f^;Rb1^f/f^*) and human prostate tumors. Transcriptomic analyses revealed special changes in the expression of genes related to the SLIT/ROBO neural signaling pathway. We further characterized the gene and protein expression of the SLIT/ROBO pathway in knockout animal samples, and protein expression in the PCa samples of patients with different Gleason scores. Public datasets with clinical data from patients (The Human Protein Atlas, cBioPortal, SurvExpress and CamcAPP) were used to validate the gene and protein expression of SLIT1, SLIT2, ROBO1, and ROBO4, correlating these alterations with the prognosis of subgroups of patients. Our findings highlight potential biomarkers of the SLIT/ROBO pathway with prognostic and predictive value, as well as promising therapeutic targets for PCa.

## 1. Introduction

Prostate cancer (PCa) accounts for approximately 7.3% of all new cancer cases and 3.8% of cancer-related deaths among men globally, making it the second most commonly diagnosed cancer and the second leading cause of cancer death in men worldwide [1]. According to the World Health Organization (CANCER TODAY), an estimated 1,467,854 new cases and 375,304 deaths were reported in 2020 alone. Alarmingly, global projections suggest that by 2030, the number of new PCa cases could rise to 1.7 million, with nearly 500,000 deaths expected [1].

Advancements in diagnostic approaches and patient risk stratification for PCa, including liquid biopsies and exosomes, have recently been described [2,3]. However, distinguishing between aggressive and indolent forms of PCa, particularly in patients with low Gleason scores, remains a significant challenge [4]. There is a critical need for new targets to better predict patient prognosis.

SLITs are secreted glycoproteins that bind to single-pass transmembrane receptors of the Roundabout (ROBO) family [5,6,7]. Members of the SLIT/ROBO family are important regulators of the embryological development of the nervous system, specifically in axon guidance. SLITs and ROBOs act as short-range repellents, controlling the crossing of axons across the midline and preventing their migration to incorrect locations [6,8,9,10]. It is now known that members of the SLIT/ROBO family also regulate proliferation, cell adhesion, and survival in various tissues, and are involved in organogenesis, angiogenesis, and tumor progression [9,11]. Studies have suggested that the guidance activity requires syndecan heparan sulfate proteoglycans at the cell surface to promote the binding of mammalian SLIT/ROBO homologues [10], with syndecans being linked to more aggressive forms of PCa and worse prognosis [12].

The ROBO receptors act as regulators of the identity of pancreatic progenitor cells. ROBO1 and ROBO2 are expressed in these cells from the time of target specification, and SLIT3 is expressed in the surrounding mesenchyme [13]. Despite limited research on the SLIT/ROBO pathway in PCa, a recent study showed that the expression of ROBO4 was associated with higher tumor histological grade; however, patients with high ROBO4 expression exhibited lower biochemical recurrence, possibly reflecting a protective role for ROBO4 [14]. Based on transcriptomic analysis (RNA-seq) of prostate cancer (PCa) samples from *Pten* and *P53/Rb1* transgenic mouse models, we focused our investigation on the SLIT/ROBO axonal guidance signaling pathway. This targeted approach was informed by prior studies suggesting a role for SLIT/ROBO signaling in cancer progression and tumor-stroma interactions. Instead of conducting an unbiased, genome-wide search for top differentially expressed genes, we opted for a hypothesis-driven strategy centered on this pathway due to its emerging relevance in PCa. The primary aim of this study was to characterize the tissue-specific localization of SLIT and ROBO proteins in PCa samples from transgenic mice and human tumors, and to explore the association between SLIT/ROBO pathway components and patient survival in prostate cancer.

## 2. Results

### 2.1. Analysis of Gene Expression of the SLIT/ROBO Pathway in Prostate Tumors from Knockout Mice

**Figure 1:**
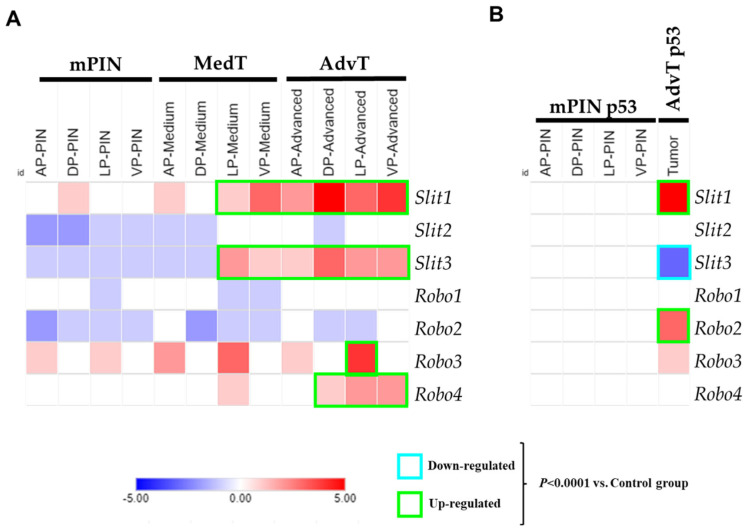
Heatmaps depicting differential expression of *Slit1*, *Slit2*, *Slit3*, *Robo1*, *Robo2*, *Robo3* and *Robo4* genes across different prostatic lobes, mouse models, and tumor stages. (**A**) Pten mouse model (**B**) P53/Rb1 mouse model. The heatmaps represent Log_2_FC of normalized RPKM values. **mPIN**—mouse Prostatic Intraepithelial Neoplasia; **MedT**—Medium-stage tumor (micro-invasive adenocarcinoma); **AdvT**—Advanced-stage tumor (invasive adenocarcinoma); **mPIN p53**—Prostatic Intraepithelial Neoplasia in P53/Rb1 mouse; **AdvT p53**—Advanced-stage tumor in P53/Rb1 mouse. **AP**—Anterior Prostate; **DP**—Dorsal Prostate; **LP**—Lateral Prostate; **VP**—Ventral Prostate. Significant upregulation of *Slit1*, *Slit3*, *Robo3* and *Robo4* in the Pten model (*p* < 0.0001 vs. control group), and significant upregulation of *Slit1* and *Robo2*, with downregulation of *Slit3* in advanced tumor from the P53/Rb1 mouse (*p* < 0.0001 vs. control group). Values between −5 and 0 represent downregulated genes (blue gradient) and between 0 and 5 upregulated genes (red gradient).

Based on analysis of the PCa transcriptome of the Pten and P53/Rb1 mouse models, we identified the SLIT/ROBO axonal guide signaling pathway. Gene expression quantified by RNA-seq demonstrated that *Slit1* and *Robo2* were upregulated in advanced PCa of the p53/Rb model while the *Slit3* gene was downregulated. In contrast, the Pten model showed significant upregulation of *Slit1*, *Slit3*, *Robo3* and *Robo4* genes (Figure 1A,B).

### 2.2. IHC Analysis of SLIT/ROBO Pathway Proteins in Prostate Tissues from Knockout Mice

**Figure 2:**
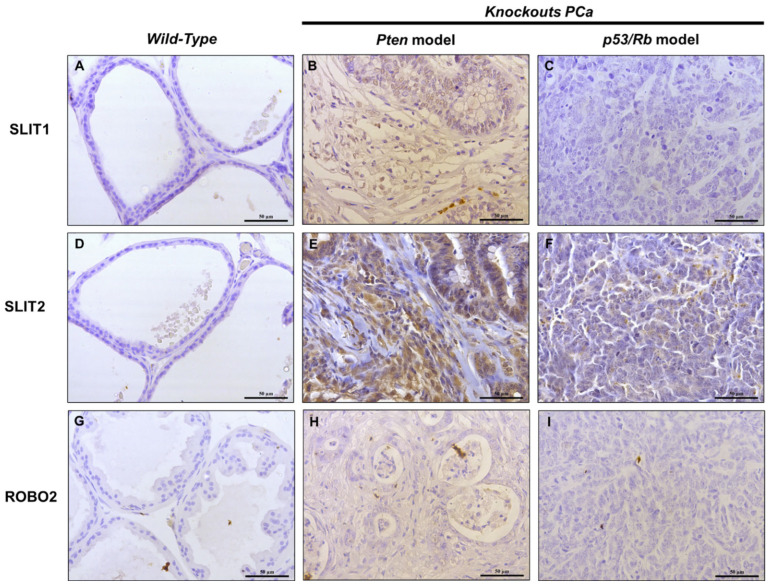
Representative photomicrographs of immunohistochemical reactions for SLIT1, SLIT2 and ROBO2 in prostatic lobes of knockout mice models (Pten and p53/Rb1). Panels (**A**–**C**) show SLIT1 expression, (**D**–**F**) show SLIT-2 expression, and (**G**–**I**) show ROBO2 expression. Wild type: Images (**A**,**D**,**G**). Advanced Pten tumors: (**B**,**E**,**H**). Advanced P53/Rb1 mouse tumors: (**C**,**F**,**I**). Scale bar corresponds to 50 μm.

We performed IHC analysis of SLIT1, SLIT2, and ROBO2 proteins in tissue samples from Pten and P53/Rb1 mouse models to verify their expression and localization in normal and tumor tissues. In WT mice, no reaction was detected for SLIT1, SLIT2 and ROBO2. In the Pten model, we observed low staining for SLIT1 in advanced-stage tumors, high-intense staining for SLIT2, and low staining for ROBO2. In the P53/Rb1 model, SLIT2 showed moderate staining in advanced tumors, while no reaction was detected for SLIT1 or ROBO2 (Figure 2). As we can see in our results, SLIT1 and SLIT2 are more expressed in the PTEN model, which presents a more advanced tumor characterized by stromal invasion.

As previously described by Santos et al. (2021) [12] and Jurmeister et al. (2018) [15], these two genetically engineered mouse models (GEMMs) of prostate cancer (PCa) exhibit distinct histopathological features and tumor progression stages. The *Pb-Cre4/Pten^f/f^* model (Pten mouse) develops mouse prostatic intraepithelial neoplasia (mPIN) lesions that progress to intermediate-stage tumors (MedT), characterized by high-grade PIN with microinvasion and prominent reactive stroma in most glandular structures. In more advanced stages, this model displays larger, heterogeneous areas of fully invasive adenocarcinoma—both well- and poorly differentiated—also associated with reactive stroma, but rarely exhibiting metastasis. These are referred to as advanced-stage tumors (AdvT) [16,17].

In contrast, the *Pb-Cre4/Trp53^f/f^*-;*Rb1^f/f^* model (*P53/Rb1* mouse) initially presents low-grade PIN lesions that do not progress through typical adenocarcinoma stages. Instead, this model develops invasive and metastatic tumors characterized by undifferentiated neuroendocrine prostate cancer (NEPC), a highly aggressive and castration-resistant PCa subtype. Details of the histopathological analyses of these knockout mice are provided in Supplementary Figure S3 (published by Santos et al., 2021 [12]).

### 2.3. IHC Analysis of SLIT/ROBO Pathway Proteins in Human Prostate Tissues

**Figure 3:**
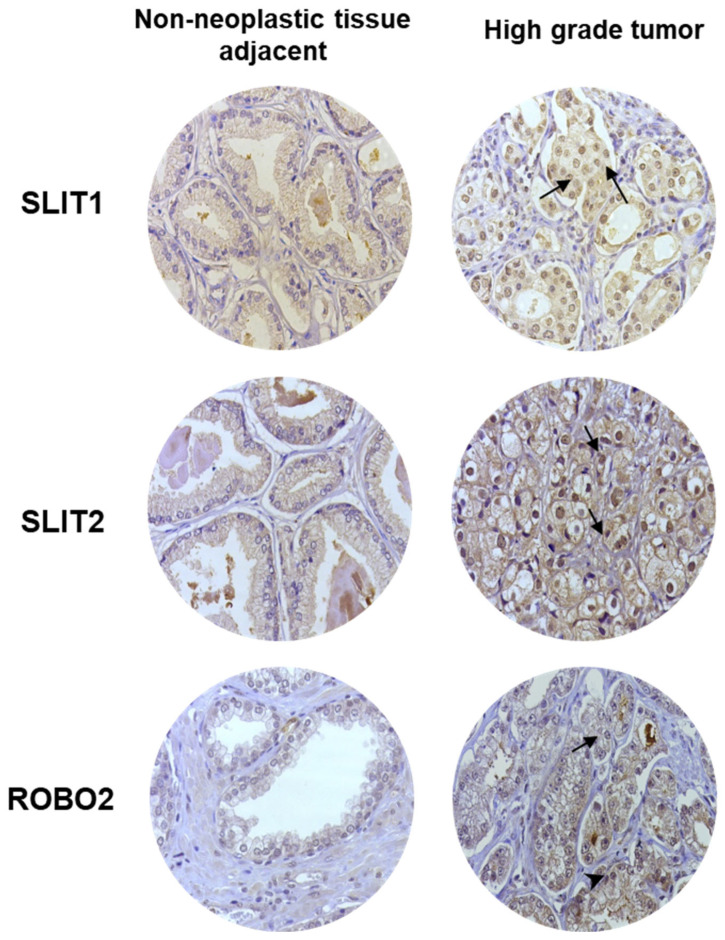
Representative images of positive immunohistochemistry reaction for SLIT1, SLIT2, and ROBO2 in normal and tumor prostate tissue samples from patients in TMAs. ×400 magnification. Arrows indicate protein expression; arrowheads indicate unmarked areas in the stroma.

We performed IHC to analyze the expression and localization of SLIT1, SLIT2, and ROBO2 proteins in human PCa and non-neoplastic samples adjacent to the tumor. The TMAs showed increased immunostaining (medium to high intensity) in PCa tumor cells, whereas non-neoplastic samples adjacent to the tumor exhibited low or undetectable levels of staining (Figure 3). No positive reactions were detected in the PCa stroma, vessels or nerve structures.

### 2.4. Prognostic Value by Gene Expression Patterns in Public Datasets

**Figure 4:**
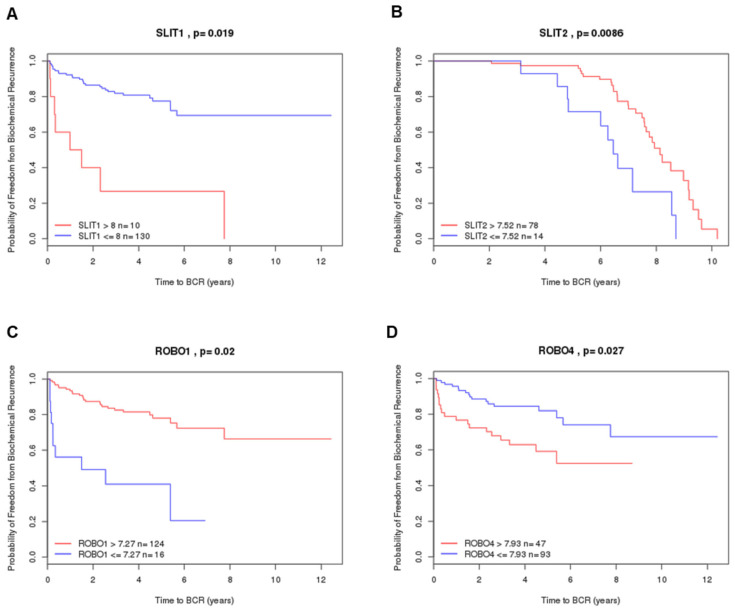
Kaplan–Meier curves displaying the probability of freedom from biochemical recurrence (BRC) of altered (red) or unaltered (blue) *SLIT1* (**A**), *SLIT2* (**B**), *ROBO1* (**C**), *ROBO4* (**D**) genes in patients with PCa. Analyzed by Cambridge Carcinoma of the Prostate App (CamcAPP) (https://bioinformatics.cruk.cam.ac.uk/apps/camcAPP/) [20] (accessed on 4 March 2025) from public datasets. (**A**)—Kaplan–Meier curve showing the probability of biochemical recurrence-free survival in patients with PCa with (red) or without (blue) *SLIT1* alterations in the MSKCC study [18]. The difference was statistically significant (*p* = 0.019). (**B**)—Kaplan–Meier curve showing the probability of biochemical recurrence-free survival in patients with PCa with (red) or without (blue) *SLIT2* alterations in the Stockholm study [21], with a statistically significant difference (*p* = 0.0085). (**C**)—Kaplan–Meier curve showing the probability of biochemical recurrence-free survival in patients with PCa with (red) or without (blue) *ROBO1* alterations in the MSKCC study [18] with a statistically significant difference (*p* = 0.02). (**D**)—Kaplan–Meier curve showing the probability of biochemical recurrence-free survival in patients with PCa with (red) or without (blue) *ROBO4* alterations in the MSKCC study [18], with a statistically significant difference (*p* = 0.027).

We analyzed the association between the expression of *SLIT1*, *SLIT2*, *ROBO1* and *ROBO4* genes and prognosis in patients with PCa using three public datasets (MSKCC, Cambridge, and Estocolmo). *SLIT1* overexpression was associated with a lower probability of freedom from biochemical recurrence in the MSKCC dataset (*p* = 0.019) (Figure 4A). In contrast, *SLIT2* and *ROBO1* in the altered group were associated with a good prognosis for biochemical recurrence in the Stockholm and MSKCC datasets (*p* = 0.0085 for *SLIT2* and *p* = 0.02 for *ROBO1*) (Figure 4B,C). *ROBO4* expression in the altered group of patients with PCa was associated with a lower probability of free biochemical recurrence in the MSKCC dataset (*p* = 0.027) (Figure 4D). The other genes (*SLIT3*, *ROBO2*, and *ROBO3*) did not show statistically significant differences in the Kaplan–Meier curves for the probability of biochemical recurrence-free (BRC) and overall survival in PCa on the CamcAPP.

**Figure 5:**
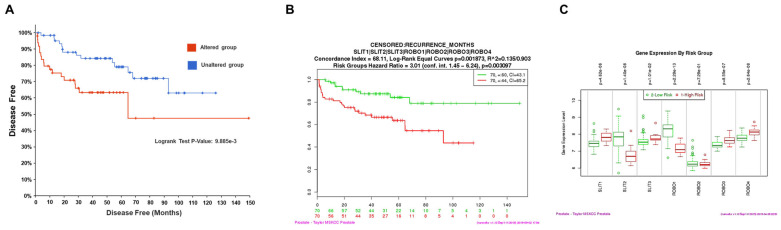
Kaplan–Meier curves showing survival analysis using cBioPortal and SurvExpress databases for SLIT/ROBO pathway genes (*SLIT1*, *-2*, *-3* and *ROBO1*, *-2*, *-3*, *-4*). (**A**)—Disease-free status in patients with prostatic adenocarcinoma, comparing gene clusters with (red) and without (blue) alterations in SLIT/ROBO genes. Data and analysis were cataloged using the cBioPortal platform from the MSKCC study [18]. (**B**)—Kaplan–Meier curve showing survival of low-risk (green) and high-risk (red) PCa patients based on SLIT/ROBO gene alterations, cataloged using the Survexpress database [22] from the MSKCC prostate study [18]. (**C**)—Expression levels (median) of SLIT/ROBO pathway genes in low-risk (green) and high-risk (red) PCa patients. Data and analysis were cataloged using the Survexpress database [22] from the MSKCC PCa study [18].

We performed the gene survival analysis of *SLIT1*, *-2*, *-3* and *ROBO1*, *-2*, *-3*, *-4* using cBioPortal for Cancer Genomics and SurvExpress Kaplan–Meier curves, based on data from the MSKCC study by Taylor et al., 2010 [18]. Survival curves showed that patients with altered SLIT/ROBO genes had significantly lower chances of being free of PCa (*p* = 9.885 × 10^−3^) (Figure 5A) and shorter time free of biochemical recurrence (*p* = 0.003097) (Figure 5B). Additionally, gene expression analysis revealed that *SLIT1*, *-2*, *-3* and *ROBO1*, *-2*, *-3*, *-4* were differentially expressed in low-risk (green) and high-risk patients (red) patients with PCa. *SLIT2*, *ROBO1* and *ROBO2* were downregulated in the high-risk patient group (Figure 5C).

Figure S1 shows the Kaplan–Meier curves for individual genes (*SLIT1*, *-2*, *ROBO1*, *-3*, and *-4*) from the MSKCC study, where patients with altered genes had a lower percentage of free PCa. Gene expression data from the SurvExpress database revealed differences between low-risk (green) and high-risk (red) patients of PCa from the TCGA study [19]. The analysis highlighted *SLIT1*, *ROBO1*, and *ROBO2* as being associated with high-risk patients (Figure S2).

## 3. Discussion

In this study, we identified the SLIT/ROBO axonal guide-signaling pathway in PCa through transcriptome analyses of prostate tissues from Pten mouse and P53/Rb1 knockout mouse models. These models show tumor progression patterns similar to those seen in human PCa, including prostatic intraepithelial neoplasia (PIN), micro-invasive and invasive well-differentiated adenocarcinoma (mid-stage tumors), and fully invasive poorly differentiated adenocarcinoma (advanced-stage tumors). Additionally, deletions and mutations in the tumor suppressor genes *Pten*, *Tp53*, and *Rb1* are among the most common genomic alterations in human PCa. These genes have been consistently associated with more aggressive disease and worse prognosis [12,23].

We found that the genes *Slit1*, *Slit3*, *Robo2* and *Robo4* were significantly altered at different stages of PCa progression, with higher expression observed in more advanced stages. Correspondingly, IHC revealed that proteins from the SLIT/ROBO pathway were present in both advanced tumors from knockout mice and human prostate tumor tissues. In our study, we observed increased expression of SLIT1 and SLIT2 in the Pten model, which develops advanced-stage adenocarcinomas with prominent stromal invasion. This finding suggests that SLIT proteins may be actively involved in the crosstalk between tumor epithelial cells and the surrounding stromal microenvironment, potentially influencing tumor architecture and invasiveness. Given that the P53/Rb1 model progresses toward an undifferentiated neuroendocrine phenotype with lower SLIT expression, these results support the idea that SLIT/ROBO signaling may have subtype-specific roles in prostate cancer and could be more relevant in adenocarcinomas with preserved stromal architecture compared to neuroendocrine tumors.

Furthermore, the gene expression profiles of these markers are altered in human PCa patients with worse overall survival and increased risk of biochemical recurrence in PCa patients. *SLIT1* and *ROBO4* had the lowest probability of freedom from biochemical recurrence. Patients with alterations in *SLIT2* and *ROBO2* genes were more likely to have no biochemical recurrence. However, patients with altered SLIT/ROBO pathway genes were collectively indicative of a worse prognosis for biochemical recurrence.

The expression of SLIT/ROBO genes has been associated with cancer prognosis and treatment response across various cancers, including ovarian cancer [24], pancreatic cancer [11,13], and breast cancer [25]. For example, the TCGA-PANCAN datasets suggest that high expressions of *SLIT1*, *SLIT2*, and *SLIT3*, coupled with low expression of one or more *ROBO* genes, may be associated with poor prognosis [26].

In PCa, a previous study by Latil et al. (2003) [27] quantified the expression of some targets of the SLIT/ROBO pathway in 48 sporadic prostate tumors using RT-PCR, but did not perform any prognostic analysis [27]. In addition to identifying the expression of all targets of the SLIT/ROBO pathway, we associated them with patient survival and tissue expression data in human PCa samples. Moreover, recent studies have demonstrated that phytochemical treatments can increase *SLIT2* and *ROBO1* expression and inhibit DU145 prostate tumor cells [28].

Although the role of the SLIT/ROBO pathway in PCa remains underexplored, its involvement in other cancers provides useful insights [13,29,30,31]. In addition to its role in axonal guidance, the SLIT/ROBO pathway regulates migration and invasion through the regulation of Wnt/β-catenin signaling [32,33].

*Robo2* suppresses stromal activation in pancreatic cancer through divergent mechanisms during development (Pinho et al., 2018). In our study, *ROBO2* was expressed in human PCa but not in PCa mouse models. In normal pancreatic parenchyma, ROBO1 and ROBO2 inhibit stromal activation. However, in the epithelial compartment, Robo2 expression is lost, while ROBO1 is increased in the stroma. Loss of ROBO2 in pancreatic epithelial cells activates the production of TGF-β, while in the surrounding stroma, ROBO2 remains active in the tumor [30,31].

More recently, SLIT2 has been shown to inhibit metastasis in breast cancer by activating M1-Like phagocytic tumor-associated macrophages (M1-TAMs), and high SLIT2 expression correlates with better patient survival [34]. Similarly, in this study, in silico analysis suggested that SLIT2 expression is associated with a favorable prognosis in PCa, with patients exhibiting less biochemical recurrence. However, RNA-seq analysis in mice showed lower *SLIT2* expression, despite strong protein expression in both human and mouse PCa tissues.

Interestingly, some studies have suggested that the absence of SLIT2 can improve patient prognosis. This SLIT2, secreted by surrounding fibroblasts, suppresses the tumor by inhibiting the PI3K/Akt/β-catenin pathway via the Robo1 receptor [32,34]. On the other hand, SLIT2-ROBO1 signaling promotes tumorigenesis and tumor growth in intestinal cancer by activating Src signaling and downregulates Wnt/β-catenin signaling [35].

Additionally, SLIT genes have been shown to be hypermethylated in gastric cancer. During early gastric tumor progression, methylation of CpG islands inactivates *SLIT1*, *SLIT2* and *SLIT3* [36]. A recent study reported that methylation in the *SLIT2* gene might be a potential biomarker for detecting non-small cell lung cancer and predicting recurrence-free survival [37], although future studies with a larger sample size are required to confirm these results.

## 4. Materials and Methods

### 4.1. Analysis of SLIT/ROBO Gene Expression in Two Genetically Modified Mouse Models (GEMM) of PCa: Pb-Cre4/Pten^f/f^ and Pb-Cre4/Trp53^f/f^-;Rb1^f/f^

RNA sequencing (RNA-seq) data and prostate samples from various stages of tumor development and progression were obtained from two GEMM of PCa, *Pb-Cre4/Pten^f/f^* model (Pten mouse) and *Pb-Cre4/Trp53^f/f^*-;*Rb1^f/f^* model (P53/Rb1 mouse). Few studies have combined tumor progression stages with all four prostatic lobes (anterior, ventral, lateral, and dorsal) in deep RNA sequencing experiments. Further details regarding these conditional *knockout* mice, histopathological analysis, and transcriptome data have been described previously [12,15,38,39].

RNA sequencing data from all four prostatic lobes were retrieved from the NCBI GEO platform (GEO, https://www.ncbi.nlm.nih.gov/geo/, accessed on 15 July 2021), reference number GSE94574. A total of 93 samples were used for RNA-seq analysis, including 20 wild-type (WT) prostatic lobes, 32 PIN-stage tumors, 20 middle-stage tumors, and 21 advanced-stage tumors. At least four samples from each prostatic lobe and pathological condition for each mouse were included. Details of this procedure have been published previously [12,15].

The animal experiments in this project were approved by the Ethics Committee of the CRUK Institute of the University of Cambridge (UK Home Office Project License 80/2435) and the Animal Experimentation Ethics Committee of the Institute of Biosciences of Botucatu (Protocol CEEA 613 /2014 and 4921200721).

For the two models, animals aged 4 to 17 months, exhibiting PIN, microinvasive adenocarcinoma, and invasive adenocarcinoma, were used in the study. Prostate tissue samples from the ventral prostate (VP), lateral prostate (LP), dorsal prostate (DP), and anterior prostate (AP) lobes of control and knockout mice were collected for RNA extraction, quantification, qualification, and sequencing. The minimum number of samples used per experimental group was four, with some tumor stages containing five samples [12,15,23].

### 4.2. Immunohistochemistry (IHC) in Mice Sample Analysis

Paraffin blocks were obtained from all prostate lobes, with WT prostate samples and tumor samples from the GEMM models provided by David Neal’s Uro-Oncology Group at the Cambridge Institute CRUK (University of Cambridge, UK). Over 10 different urogenital complex paraffin blocks from WT mice and 20 blocks from both knockout mouse models were sectioned. Prostate histological sections at different stages of development and progression were deparaffinized, hydrated, and washed in PBS (0.1 M, pH 7.4). Antigen retrieval was performed using 10 mM citrate buffer (pH 6.0) for 35 min in a Dako pressure cooker. The sections were then treated with a 3% H_2_O_2_ solution in methanol for 10 min to block endogenous peroxidase activity. To block protein–protein interactions, sections were incubated in 3% BSA in PBS before overnight incubation at 4 °C with primary antibodies against SLIT1 (PA5-119421, Invitrogen, Waltham, MA, USA), SLIT2 (MA5-42813, Invitrogen, Waltham, MA, USA) or ROBO2 (PA5-113491, Invitrogen, Waltham, MA, USA), all diluted 1:100 in 1% BSA solution in PBS.

Antibodies were purchased from Thermo Fisher (Waltham, MA, USA). The slides were washed with PBS, incubated with peroxidase-conjugated secondary antibodies and exposed to diaminobenzidine as a chromogen. Finally, sections were stained with hematoxylin. Images of the slides were captured using a Leica DM2500 microscope and acquired using a Leica DMC2900 camera (Leica Qwin Image Analysis Software version 3.1.)

In IHC analysis, a descriptive approach was adopted based on the visual assessment of staining intensity (categorized as low, moderate, or high) and the distribution patterns across different tumor stages and mouse models.

### 4.3. Clinical Information of Patients with Prostatic Carcinoma Used for the Construction of Tissue Microarrays (TMAs)

Paraffin-embedded PCa samples were obtained from patients treated at the Hospital of the Medical School of Botucatu, Department of Pathology, FMB/UNESP. Clinical data for patients diagnosed with prostatic adenocarcinoma at the Clinical Hospital of the Medical School of Botucatu (FMB) were surveyed. Paraffin blocks, available in the Department of Pathology at FMB from 1980 to 2000, were used to create tissue microarray (TMA) blocks.

A total of 119 suitable cases were selected for analysis and subsequent immunohistochemistry (IHC) studies. TMA blocks were prepared with two neoplasm samples from each patient. These PCa samples from the 119 patients were organized into TMAs and subjected to IHC using the protocol described in subtopic 2.2 to evaluate the tissue expression of SLIT1, SLIT2, and ROBO2 proteins in human PCa.

The histopathological analysis of these tumors was reviewed by two pathologists, and the prostatic adenocarcinomas were reclassified according to the recent criteria established by the WHO Tumor Classification and the International Society of Urological Pathology (ISUP) [40]. The samples were classified as Gleason 6 (3 + 3), Gleason 7 (3 + 4), Gleason 8 (3 + 5), Gleason 7 (4 + 3), Gleason 8 (4 + 4), Gleason 9 (4 + 5), Gleason 8 (5 + 3), Gleason 9 (5 + 4), and Gleason 10 (5 + 5). They were grouped into prognostic categories established by ISUP as follows: Gleason 6 (3 + 3) corresponds to prognostic category 1; Gleason 7 (3 + 4) to category 2; Gleason 7 (4 + 3) to category 3; all cases with a Gleason score of 8 or higher to category 4; and cases with Gleason 9 and 10 to category 5. This study was approved by the Medical Ethics Committee of FMB/UNESP (Protocol No. 3888/2011).

### 4.4. Prognostic Value Analysis in Public Datasets

The expression of SLIT/ROBO pathway genes was investigated using published PCa datasets. Gene expression patterns were analyzed using the SurvExpress database [22], the Cambridge Carcinoma of the Prostate App (CamcAPP) database developed by the Cancer Research UK Cambridge Institute [20] (https://bioinformatics.cruk.cam.ac.uk/apps/camcAPP/) (accessed on 4 March 2025), cBioPortal for Cancer Genomics [41,42] (https://www.cbioportal.org/) (accessed on 4 March 2025), and The Cancer Genome Atlas (TCGA) [19].

The databases were used to determine the genetic changes in patient clinical data, including survival rates and risk/prognosis. Gene expression associated with poorer prognostic outcomes was also examined in the published Cambridge dataset [21] and the MSKCC dataset [18].

### 4.5. Statistical Analysis

One-way analysis of variance (ANOVA) with Dunnett’s multiple comparison post-test was used for gene expression analysis. A survival curve was constructed using the Kaplan–Meier method and compared using the log-rank (Mantel–Cox) test. Differences were considered statistically significant when *p* ≤ 0.05. Statistical analyses were performed using GraphPad Prism software version 8.0 (San Diego, CA, USA).

## 5. Conclusions

In this study, we demonstrated that the expression of SLIT/ROBO pathway genes varies across different models and stages of PCa progression. The expression patterns at different stages of prostate tumors change according to the genetic heterogeneity of the tumors. Our findings suggest that SLIT2 expression may be associated with a better prognosis for patients, as it is not highly expressed in mouse models, and its alteration correlates with a shorter time to biochemical recurrence in patients. Therefore, SLIT2 expression could serve as a valuable prognostic marker for PCa, which may contribute to improved patient stratification and personalized therapies for PCa.

## Data Availability

The results presented in this article are partly based on data generated by the Cambridge Carcinoma of the Prostate App (CamcAPP) (https://bioinformatics.cruk.cam.ac.uk/apps/camcAPP/) (accessed on 4 March 2025) from the Cancer Research UK Cambridge Institute, cBioPortal for Cancer Genomics database (https://www.cbioportal.org/) (accessed on 4 March 2025), and Human Protein Atlas (HPA) (https://www.proteinatlas.org/) (accessed on 4 March 2025) database. The RNA sequencing data derived from all prostatic lobes of Pten-knockout mice are available in the NCBI Gene Expression Omnibus platform (GEO, https://www.ncbi.nlm.nih.gov/geo/) (accessed on 4 March 2025) (reference number GSE94574).

## Supplementary Materials

The following supporting information can be downloaded at: https://www.mdpi.com/article/10.3390/ijms26115265/s1.
