# Supplementary material for: SLIT/ROBO Pathway and Prostate Cancer: Gene and Protein Expression and Their Prognostic Values
Source: Int J Mol Sci. 2025 May 30;26(11):5265. doi: 10.3390/ijms26115265 (PMC12155087; doi:10.3390/ijms26115265)
Supplement: Supplementary file 1 [file ijms-26-05265-s001.zip › ijms-3616491-supplementary.pdf]

# Supplementary Figures

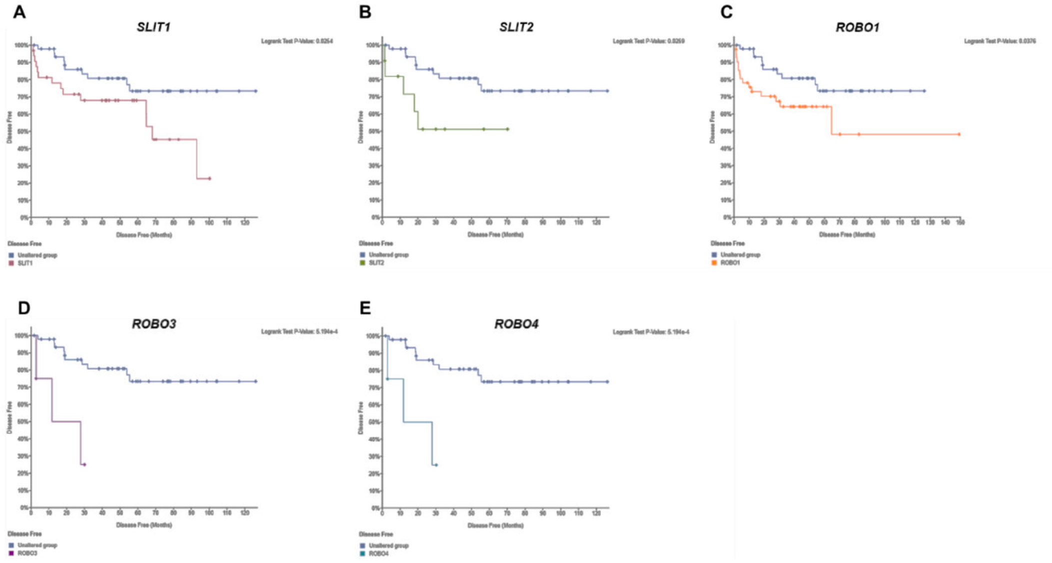


**Figure S1.** Kaplan-Meier curves showing individual survival analysis for *SLIT1* (**A**), *SLIT2* (**B**), *ROBO1* (**C**), *ROBO3* (**D**), *ROBO4* (**E**) genes using cBioPortal databases, with disease-free status in patients with prostatic adenocarcinoma with altered (red) and unaltered (blue) genes of the SLIT/ROBO pathway. Data and analysis were cataloged using the cBioPortal platform from the MSKCC study [18]. The *SLIT3* and *ROBO2* genes did not show significant survival differences in this analysis. P value < 0.05.

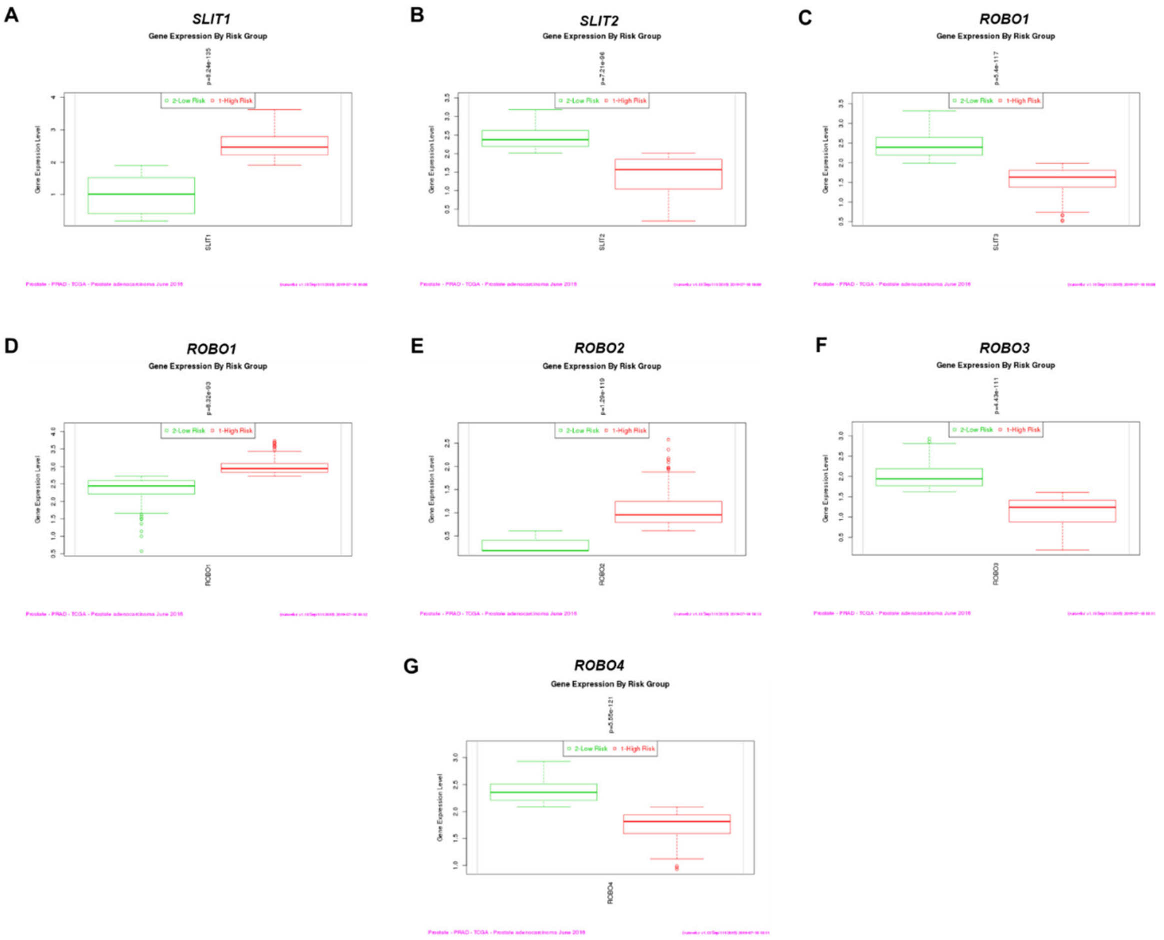

**Figure S2.** Gene expression of genes *SLIT1* (**A**), *SLIT2* (**B**), *SLIT3* (**C**), *ROBO1* (**D**), *ROBO2* (**E**), *ROBO3* (**F**), *ROBO4* (**G**), with expression level (median) of SLIT/ROBO pathway genes in low-risk (green) and high-risk (red) PCa patients. Data and analysis were cataloged using the Survexpress database [22] from the TCGA. *SLIT1*, *ROBO1* and *ROBO2* genes were upregulated in the high-risk group (red), while *SLIT2*, *SLIT3*, *ROBO3* and *ROBO4* genes were downregulated in the high-risk patients and upregulated in the low-risk group. P value $< 0.05$.

18. Taylor, B.S., et al., *Integrative genomic profiling of human prostate cancer.* Cancer Cell, 2010. **18**(1): p. 11-22.
22. Aguirre-Gamboa, R., et al., *SurvExpress: an online biomarker validation tool and database for cancer gene expression data using survival analysis.* PLoS One, 2013. **8**(9): p. e74250.
